# Supplementary material for: Aptamer-Gated Mesoporous Silica Nanoparticles for N Protein Triggered Release of Remdesivir and Treatment of Novel Coronavirus (2019-nCoV)
Source: Biosensors (Basel). 2022 Nov 1;12(11):950. doi: 10.3390/bios12110950 (PMC9688528; doi:10.3390/bios12110950)
Supplement: Supplementary file 1 [file biosensors-12-00950-s001.zip › biosensors-1969869-supplementary.pdf]

biosensors

MDPI

Supporting Information

Aptamer-Gated Mesoporous Silica Nanoparticles for N Protein Triggered Release of Remdesivir and Treatment of Novel Coronavirus (2019–nCoV)

Xiaohui Zhang [1], Xin Zhang [1], Aoqiong Xu [1], Mengdi Yu [1], Yu Xu [1], Ying Xu [1], Chao Wang [1], Gege Yang [1], Chunxia Song [1,2], Xiangwei Wu [3,*] and Ying Lu [1,2,*]

1 Department of Applied Chemistry, Anhui Agricultural University, Hefei 230036, China
2 Key Laboratory of Agricultural Sensors, Ministry of Agriculture and Rural Affairs, Anhui Agricultural University, Hefei 230036, China
3 Key Laboratory of Agri–Food Safety of Anhui Province, College of Resources and Environment, Anhui Agricultural University, Hefei 230036, China
* Correspondence: luy@ahau.edu.cn (Y.L.); wxw@ahau.edu.cn (X.W.)

Reagents and Materials

Remdesivir (RDV; 99.74%) was acquired from MedChemExpress (Monmouth Junction, NJ, USA). N–hydroxysuccinimide (NHS), immunoglobulin G (IgG), hemoglobin (HGB), fibrinogen (FIB), cytochrome C, thrombin, lysozyme (LZ) and 50 × TAE were acquired from Beijing Soleibo Technology Co., Ltd. Nucleocapsid protein (N protein) was purchased from Beijing Yiqiao Shenzhou Technology Co., Ltd. Marker, 6 × Loading buffer, Agrose and T4 polynucleotide kinase (T4 PNK) was bought from Sangon Biotechnology Inc. (Shanghai, China). Bovine serum albumin (BSA) was acquired from Shandong sikejie Biological Co., Ltd. Alkaline phosphatase (AKP) was supplied by Shanghai Biyuntian Biological Co., Ltd. SYBRTM Gold nucleic acid gel stain was from Thermo Fisher scientific corporation. N protein aptamers and complementary DNA chain were synthesized and purified by Sangon Biotechnology Inc. All the solutions were prepared with ultrapure water obtained from a Millipore water purification system (>18.2 MΩ·cm). All other chemicals were of analytical grade.

Instrumentation

Ultraviolet–visible (UV–vis) absorption spectra were recorded with a TU–1901 UV–vis spectrophotometer (Beijing). Scanning Electron Microscope (SEM) image was obtained by using Hitachi S–4800 (Japan). X-ray diffraction (XRD) image was obtained by using Bruker D8 Advance (Bruker Germany). The concentration of N protein aptamer and complementary chain was obtained by Nucleic acid quantizer **(NANODROP 2000 America)**. The synthesis of DNA double strands was performed by Polymerase Chain Reaction **(PCR; Hermal Cycler S1000 America)**. Electrophoresis bands were formed by a DNA electrophoresis instrument (JY300C China). Gel imaging was obtained by Analyzing instrument of formation of image of gel (ChemiDOC MP imaging system America).

Experimental Section

Synthesis of ds–DNA Required for DNA Electrophoresis.

The synthetic and quantitatively analyzed N protein aptamers were hybridized with complementary DNA to form double-stranded DNA (ds–DNA) oligonucleotides by mixing N protein aptamers (1 μM) and complementary DNA (1 μM) into 43 μL of Tri–$MgCl_2$ (5 mM $MgCl_2$, 140 mM NaCl, 20 mM Tri–base pH 7.6), and 5 μL the annealing buffer (100 mM Tris, 0.5 mM EDTA, 1 mM NaCl, pH 7.4) was added. Then, the entire system was heated at 95 °C for 10 min and gradually cooled to 20 °C at a rate of 1 °C / min.

Gel Electrophoresis

The samples were characterized using 3% agarose gel electrophoresis in a 1 × TAE buffer at 100 V for 30 min. Then, a gel image was obtained by the analyzing instrument of formation of gel.

SEM Image

The 1 mg sample was dispersed in 2 mL anhydrous ethanol and dropped on a silicon wafer. The morphology of the sample was observed by scanning electron microscope.

RDV Release of RDV−loaded Silica over time

An MSN−Au solution of 100 μL was centrifuged with an ultrafiltration tube and cleaned by PBS to remove the excess RDV. Then, the MSN−Au was dried in a vacuum oven at 60 °C and dissolved in a 100 μL PBS buffer solution in which 2 μM of N protein was added. Another 100 μL of MSN−Au was prepared by the same process except without N protein added. The concentration of RDV in the solution was measured by UV−vis.

N protein Aptamer Selectivity

Nine common blood disruptors, i.e., AKP (20 U·mL^{-1}), BSA (2 μM), T4 PNK (20 U·mL^{-1}), IgG (2 μM), thrombin (2 μM), HGB (2 μM), LZ (2 μM), cytochrome C (2 μM), FIB (2 μM) and N protein (2 μM) were selected to evaluate the binding specificity of aptamer to N protein. The releasing of RDV was measured by detection the peak signal of UV−vis at 247 nm.

Linear Relationship between N Protein Concentration and RDV Releasing Concentration

The RDV-loaded MSN−Au solution 100 μL was centrifuged with an ultrafiltration tube and washed with PBS. Then, the MSN−Au was removed to 100 μL of PBS solution. N protein was added to the final concentration of 0.5 μM, 1 μM, 1.5 μM, 2 μM, 2.5 μM, 3 μM and 3.5 μM, respectively. The concentration of RDV release into the solution was obtained by UV−vis.

Actual Sample Spike Recovery Experiment

In order to evaluate the accuracy of this method in the detection of real samples, the average recovery rate of N protein in blood samples was obtained by using statistical methods in this experiment. Amounts of 0.5 μM, 1 μM and 2 μM of N protein were added to the blood samples, respectively, at three concentrations in parallel for each group. The ratio of the UV−vis absorption peak of RDV measured in the actual sample to the UV−vis absorption peak of RDV in the standard curve was taken as the recovery rate.

Table S1. N protein aptamer and complementary chain.

Types	Sequences of DNA (from 5′ to 3′)
N protein aptamer	5′−GCTGG ATGTC GCTTA CGACA ATATT CCTTA GGGGC ACCGC TACAT TGACA CATCC AGC−SH−3′
Complementary chain of N protein aptamer	5′−COOH−TTGTA CTGGC TCATA GCTGG ATGTG TCAAT GTAGC GGTGC CCCTA AGGAA TATTG TCGTA AGCG−3′

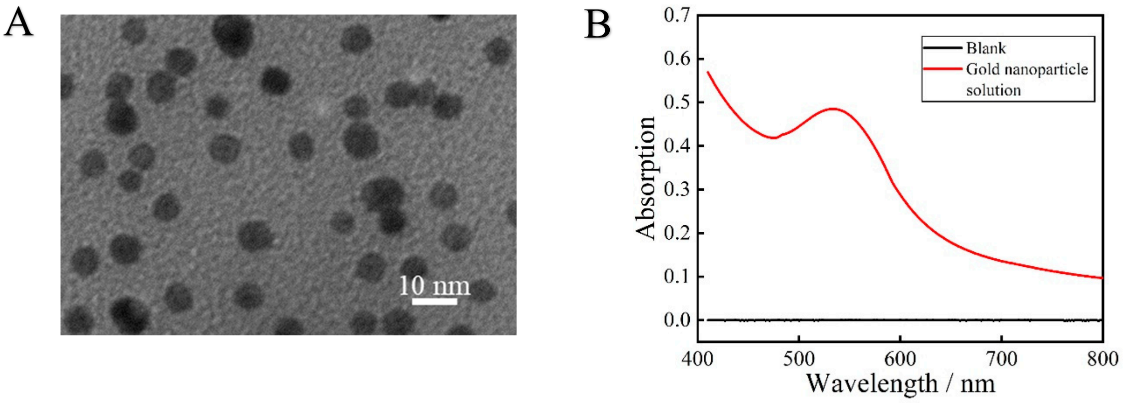

Figure S1. (A)TEM image and (B) UV−vis absorption diagram of gold nanoparticles.

The gold nanoparticles were characterized by TEM and UV−vis. Figure S1. In the TEM image (A), gold nanoparticles show average diameter in the range of 5−10 nm. As show in UV−vis absorption diagram (B), gold nanoparticles show an obvious absorption peak at about 530 nm compare with the blank solution.

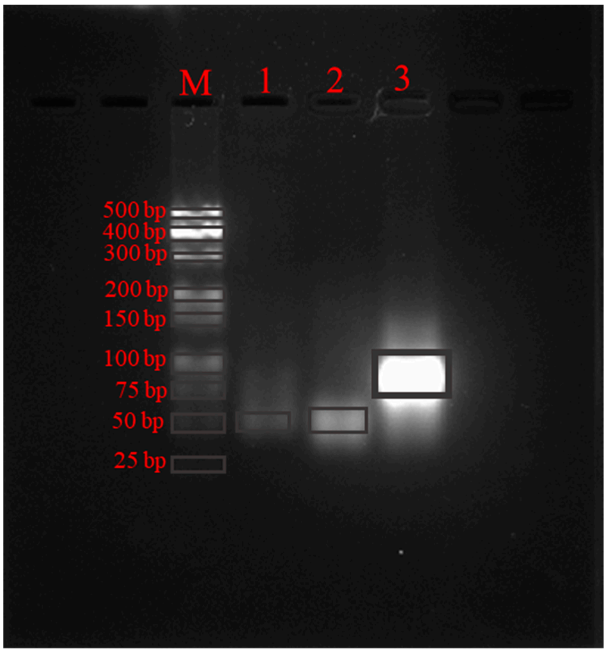

Figure S2. Agarose gel electrophoresis diagram. From left to right: mark standard strip (M), N protein aptamer (1), cDNA (2), ds−DNA (3).

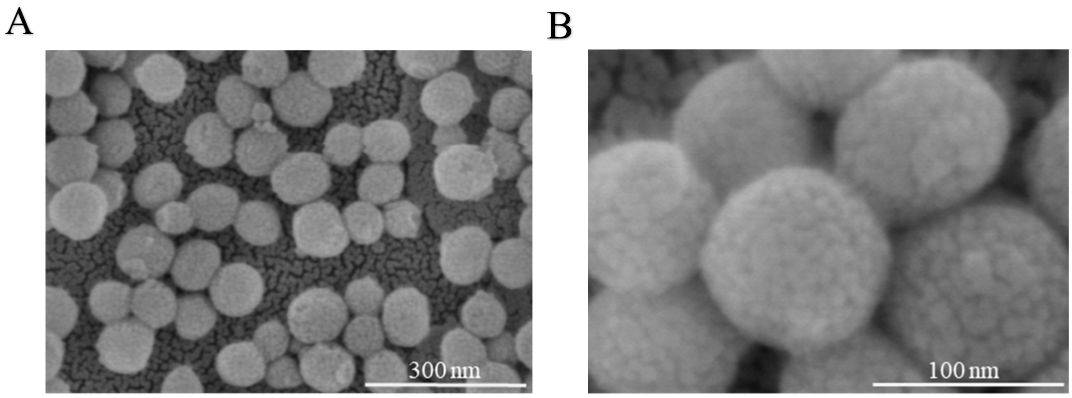

Figure S3. SEM image of mesoporous silica. Morphology characteristics of mesoporous silica under electron microscope at 150 (A) and 300 (B) magnification times.

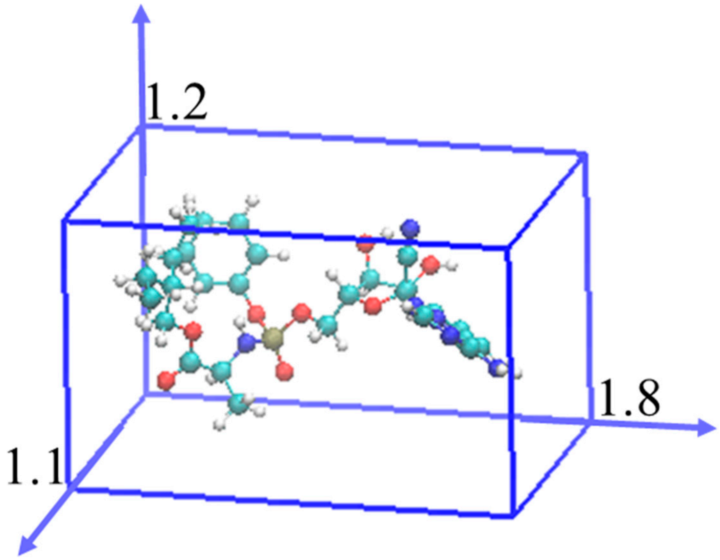

Figure S4. 3D schematic diagram of RDV. The RDV size is obtained by using the Multiwfn Version 3.7 [1].

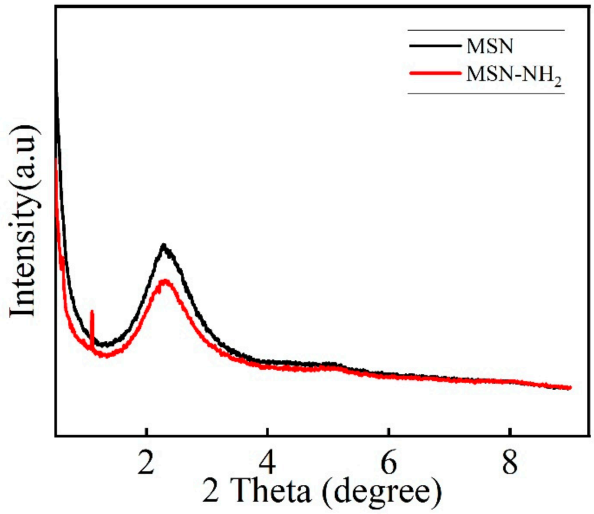

Figure S5. Powder X-ray diagram of solids MSN (black line) and MSN−NH_2 (red line).

The diffraction peak is about 2.300° at 2θ, which indicates that the materials are arranged orderly at a certain scale [2,3]. The amine-modified mesoporous silica of powder X-ray diffraction (XRD) peak is lower than that mesoporous silica of powder X-ray diffraction (XRD) peak. This might be due to the amino modified MSN material reduced the intensity of the powder X-ray diffraction (XRD) peaks of MSN [4].

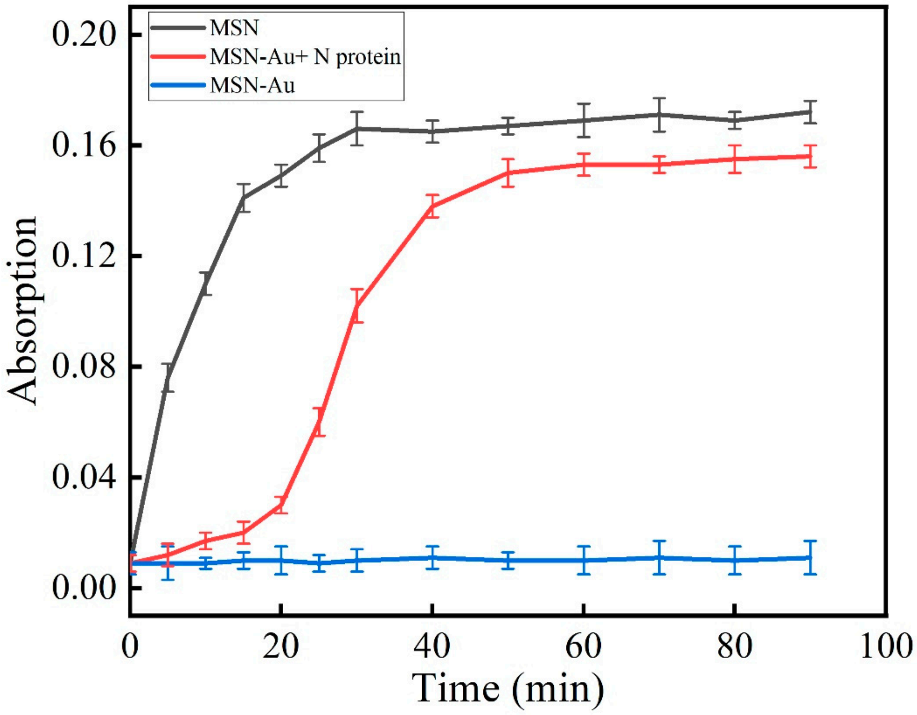

Figure S6. Optimization of time of RDV releasing from MSN−Au in the absence (blue line) and presence of 2 μM of N protein (red line). Control experiment RDV loaded mesoporous silica modified without gold nanoparticles (black line).

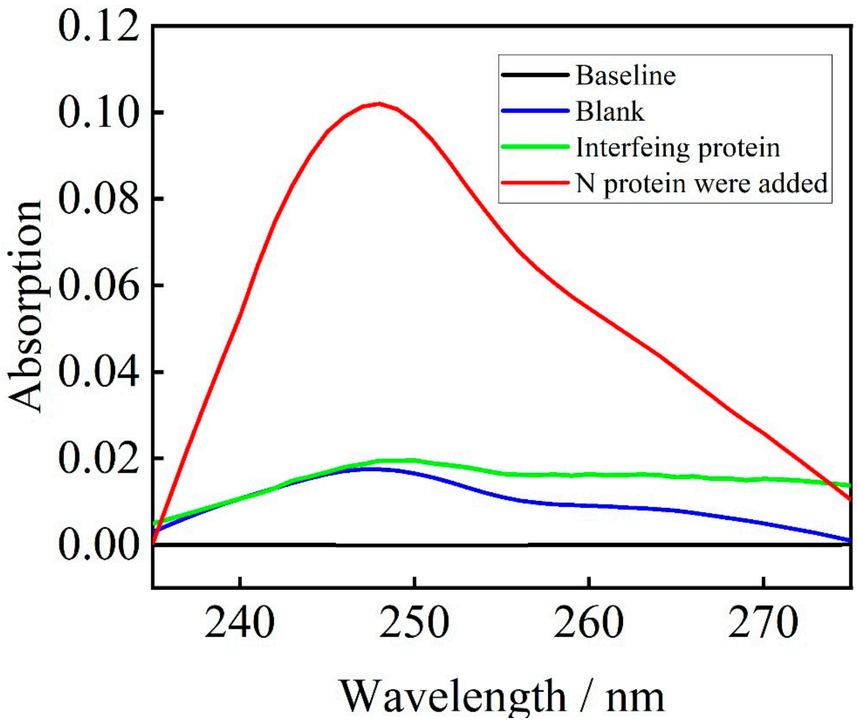

Figure S7. The UV−vis absorption peaks of RDV in the solution after the interfering substance and N protein reacted with MSN−Au solution, respectively.

In order to study the selectivity of N protein aptamer toward target, nine proteins generally found in blood were selected as interfering substances. Figure S7 shows that the UV−vis absorption peak of the concentration of RDV in the protein mixture (green line) is similar to that in the MSN−Au solution without N protein (blue line). When the N protein is added to the solution containing MSN−Au, the absorption value of UV−vis peak increases significantly (red line). It indicates that RDV was released from the mesoporous silica channel and the concentration of RDV in the solution increased. This result confirmed the specific binding property of N protein aptamer and N protein, which can be used for targeted treatment of SARS-CoV-2.

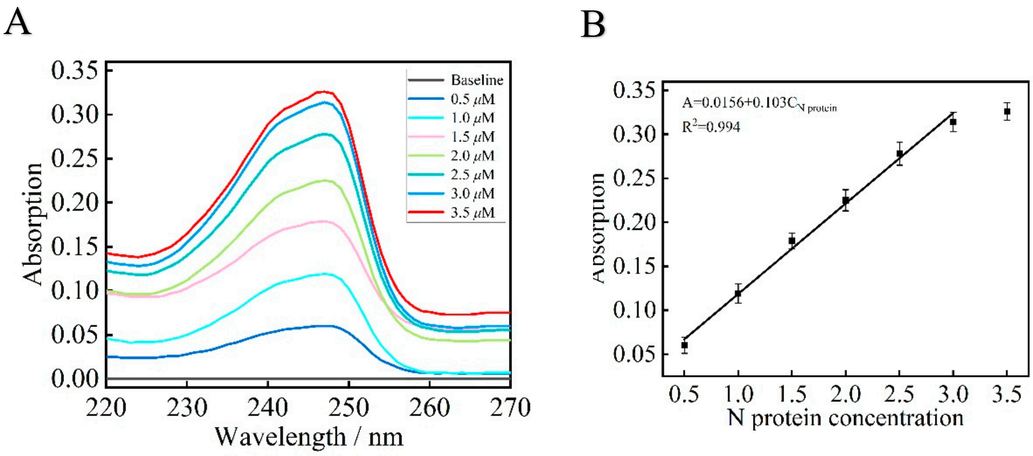

Figure S8. UV−vis spectra for different concentrations of N protein incubated with MSN−Au (A) and the correlation between the UV−vis for RDV with N protein concentration (B).

The concentration of RDV in PBS solution could be determined by UV−vis method. As shown in Figure S8A, the baseline represents the UV−vis diagram of PBS, and the concentration of N protein added from bottom to top are 0.5 μM, 1 μM, 1.5 μM, 2 μM, 2.5 μM, 3 μM, and 3.5 μM, respectively. The absorption signal intensity at 247 nm increase with the increasing concentration of the N protein. The relationship between UV−vis intensity and N protein concentration is linear in the range of 0.5–3.5 μM, and the linear regression equation is $A = 0.016 + 0.103C_{N\ protein}$, $R^2 = 0.994$ (Figure S8B). The detection limit is tested to be 0.1 μM (S/N = 3).

Table S2. Basic clinical features of the five volunteers.

Volunteers	Sex	Age	Height (cm)	Weight (kg)	Healthy
A	female	42	166	52	yes
B	male	39	183	68	yes
C	male	27	174	65	yes
D	female	24	158	44	yes
E	female	22	156	50	yes

Table S3. Recovery results of N protein induced RDV signals in human blood samples (n=3).

	Blood samples						
**$C_{N\ protein}$ (μM)**	Recovery (%)						RSD
	A	B	C	D	E	Average (%)	(%)
**0.5**	108.1	102.2	90.4	99.3	105.2	101.4	6.7
1	106.7	101.7	108.4	103.4	99.1	103.9	3.6
2	112.6	97.3	110.4	113.1	102.7	107.2	6.5

In this experiment, the average recovery rates of N protein induced RDV signals in blood samples were calculated by statistical method. The recovery experiments were through with N protein spiked at 0.5 μM, 1 μM and 2 μM in blood samples. As shown in Table S3, the obtained recovery of the blood samples was in the range of 90.4% −113.1% with RSD from 3.6% to 6.7%.

References

(1) Lu, T.; Chen, F. Multiwfn: a multifunctional wavefunction analyzer, *J. Comput. Chem.* **2012,** *33,* 580−592.

(2) Ding, H.; Zhang, Y.; Wang, S.; Xu, J.; Xu, S.; Li, G. Fe_3O_4@SiO_2 core/shell nanoparticles: the silica coating regulations with a single core for different core sizes and shell thicknesses, *Chem. Mater.* **2012,** *24,* 4572−4580.

(3) Kim, H.; Kim, T.; Roev, V.; Lee, H.; Kwon, H.; Lee, H.; Kwon, S.; Im, D. Enhanced electrochemical stability of Quasi−Solid−State electrolyte containing SiO_2 nanoparticles for Li-O_2 battery applications, *ACS Appl. Mater. Interfaces* **2016,** *8,* 1344−1350.

(4) Marler, B.; Oberhagemann, U.; Vortmann, S.; Gies, H. Influence of the sorbate type on the XRD peak intensities ofloaded MCM−41[1], *Microporous Mater.* **1996,** *6,* 375−383.
